# Workplace Safety Management Practices, Fear, Resources, and Employee Involvement During the COVID-19 Pandemic: A Narrative Review

**DOI:** 10.1016/j.focus.2025.100456

**Published:** 2025-10-06

**Authors:** Keisuke Kokubun

**Affiliations:** Graduate School of Management, Kyoto University, Kyoto, Japan

**Keywords:** COVID-19, employee involvement, fear, resources, safety compliance, safety management practices

## Abstract

**Introduction:**

There are important workplace health lessons to be learned from the pandemic.

**Methods:**

This study summarizes the relationships between workplace safety practices, fear, resources, and employee engagement during the COVID-19 pandemic through a narrative review on articles published between January 2020 and June 2025 using a primary literature search base.

**Results:**

Organizations have had to implement workplace safety management practices aligned with their occupational safety and health management systems in response to COVID-19. Safety management practices include safety initiatives and training as well as employee involvement. Methods to increase employee involvement include fear and anxiety. However, although fear and anxiety promote safety compliance and safe behavior, they also wear down employees and increase their work distraction and turnover intentions. Therefore, social and psychological resources need to be strengthened to overcome this dilemma. These resources can also help safety management practices today as the pandemic begins to wind down.

**Conclusions:**

Future research should focus on identifying ways to strengthen employees' social and psychological resources without relying on disasters. To this end, an integration of conservation of resource theory and behavioral theory may be useful.

## HIGHLIGHTS

• During the COVID-19 pandemic, fear and anxiety promote safety compliance and safety behaviors, but they also exhaust employees and lead to reduced concentration on work and increased intention to quit.
• To overcome this dilemma, it is necessary to strengthen social and psychological resources.
• Future research should focus on finding ways to strengthen employees' social and psychological resources, and the integration of conservation of resource theory and behavioral theory may be useful.
• This narrative review summarizes the relationship between workplace safety practices, fear, resources, and employee involvement during the COVID-19 pandemic.

## INTRODUCTION

Human history can be seen as a record of a continuous battle against disease.^1^^,^^2^ Therefore, organizations need to prepare effective strategies to manage current and future disease outbreaks. During periods of sustained safety threats, a safe work environment serves as the most important job resource.^3^, ^4^, ^5^ For example, workplace safety prevents the spread of disease and related accidents and injuries, preserves employment,^6^ and strengthens the positive relationship between safety knowledge and safety behaviors.^7^^,^^8^ Furthermore, a workplace safety climate may play a protective role against stressors, for example, by reducing emotional exhaustion and physical symptoms^9^ and bullying behaviors,^10^ and promote a sense of security and comfort in the workplace.^11^ During a crisis, organizations typically implement safety management practices (SMPs)—a collective term for the strategies, policies, measures, procedures and actions—in line with their occupational safety and health management systems (OSHMS) to reduce hazards and keep the workplace safe and employees healthy.^12^^,^^13^ During coronavirus disease 2019 (COVID-19) pandemic, all organizations had to focus on SMPs, especially (1) management’s commitment to safety (MCS), including the formulation of safety rules and procedures; (2) safety training; and (3) employee involvement according to the International Labor Organization.^5^^,^^14^ Employee involvement includes opportunities for all employees to discuss pandemic prevention and control, have a say in all health and safety-related issues, and be regularly consulted about workplace health and safety and is primarily intended to increase safety participation and safety compliance.^5^^,^^14^ Safety participation refers to discretionary and voluntary actions to enhance workplace safety, such as participation in safety-related programs and meetings. Safety compliance refers to activities to keep the workplace safe, such as adherence to standards and procedures specified by the organization.^15^ Employee involvement can be promoted by inducing fear and anxiety as well as through a series of SMPs implemented by the organization.^16^^,^^17^ However, excessive reliance on fear and anxiety is dangerous because they have a negative impact on employees’ health and behavior. Social and psychological resources can serve as alternatives to or alleviate fear and anxiety.^18^^,^^19^

This review looks back at the 3 elements of SMPs, discusses the pros and cons of relying on fear and anxiety to implement them, and discusses the significance of utilizing social and psychological resources. Finally, it considers ways to increase resources in preparation for the next disaster.

## METHODS

A narrative literature review on workplace SMPs, fear, resources, and employee involvement was conducted following the recommendations of Green et al.^20^ The literature search in Scopus, PubMed, Web of Science, and PsychINFO databases focused on articles published between January 2020 and June 2025 and aligned with the objectives outlined in the previous section. However, studies before 2019 were also included if deemed relevant. The keywords used were *COVID-19, employee involvement, fear, psychological resources, safety compliance, safety management practices*, and *social resources* in various combinations connected with AND OR as well as in combination with the words review or meta-analysis. The search was performed in the title and abstract. In other words, this review focused on workplace SMPs, fear, resources, and employee involvement during the COVID-19 pandemic, as titled, as well as on these general reviews. In cases where multiple reviews or articles on the same topic were identified, the most recent and/or most cited ones were given priority. In addition, the reference sections of the selected papers were examined to check for possible additional research. Also included were reports from authoritative international organizations, relevant consensus statements and documents, and empirical studies that were specifically relevant to this topic. In addition, the method used in this study was a narrative review, not a systematic review, and therefore, related literature was not covered comprehensively. For example, studies that dealt with workplace SMPs during the COVID-19 pandemic but did not discuss it from the perspective of fear, resources, and employee involvement were not included in this review.

## RESULTS

### Management's Commitment to Safety, Including the Development of Safety Rules and Procedures

MCS stands for "the extent to which management is perceived to place a high priority on safety and communicate and act on safety issues effectively" (^15^^: p. 27^). Organizations must set effective safety rules and procedures to ensure that employees can perform tasks without the risk of injury or illness in the event of a disaster. Documentation of the necessary measures will make it easier for managers to carry out safety inspections and for employees to implement safety compliance and behaviors, thereby preventing accidents and infections from occurring.^13^^,^^21^ Therefore, when the COVID-19 pandemic occurred, managers implemented MCS to reduce the risks in their organizations. Specifically, the organization urgently established a pandemic prevention committee/team to consider and develop all possible response scenarios in case of the pandemic spreading.^5^ The organization then had to strictly and effectively implement measures to prevent COVID-19 by quickly eliminating dangerous practices and other issues related to COVID-19 in the workplace. These included filling out travel history forms, checking body temperatures, applying disinfectant sprays, practicing social distancing, teleworking, and others.^5^ At the same time, organizations had to provide employees with sufficient personal protective equipment, including hand washing products, gloves, masks, and others. In parallel, managers promoted internal communication on COVID-19 prevention through newsletters, emails, and social network applications.^5^ Implementing MCS created a safe climate and reduced employees' psychological distress,^22^ while preserving their trust in the organization, making them feel more secure about their health and work, and reducing job insecurity.^15^^,^^23^^,^^24^ In particular, employees in organizations with robust response measures or those that offer permanent remote working were more likely to have good mental health and well-being.^25^ On the other hand, failure to achieve a safe environment can lead to a decline in employees’ physical and psychological health, which can lead to occupational stress.^26^^,^^27^

### Safety Training

Safety training should be provided to employees of all job types and experience levels to improve their awareness, knowledge, skills, and attitudes toward health and safety in the workplace.^5^ When management implements SMPs on communication, training, rules, and others, employees' willingness to comply with and participate in safety will be enhanced according to the social exchange theory.^28^, ^29^, ^30^ For example, the provision of training on workplace health and safety has led employees to be more positive in practicing safety compliance and participation.^5^^,^^31^^,^^32^ Therefore, organizations need to provide employees with structured and comprehensive health and safety training programs so that they know how to prevent the spread of infectious diseases, assess the current risks in the workplace in light of this knowledge, and protect themselves against these risks.^5^^,^^13^ Participation in such face-to-face or online training enabled employees to adopt appropriate behaviors to avoid COVID-19 infection, such as wearing masks and washing hands.^33^ Therefore, to be most effective, it was necessary to involve as many employees as possible in the training programs.^34^

### Employee Involvement

Employee involvement is also considered an SMP.^13^^,^^35^^,^^36^ Managers need to understand the difficulties employees are facing during the COVID-19 pandemic and create conditions for them to actively participate in the organization's disease control. Employee involvement is often expressed in the form of organizational citizenship behavior (OCB). OCB refers to actions that members take for the company or organization beyond their roles^37^ and is especially necessary during crises that require employee safety compliance and participation, such as the COVID-19 pandemic.^38^ If done properly, SMPs can reduce employee anxiety and promote employee involvement and OCB for safety compliance and participation. Employee involvement includes opportunities for discussion and consultation on pandemic prevention and control, employee health, workplace safety, and others. Providing such opportunities is crucial for employees, especially those in dangerous work environments.^39^

However, cultural and occupational differences often made it difficult to put SMPs into practice, especially employee involvement. Even during the COVID-19 lockdown, individualistic people were more likely to violate social distancing rules out of selfishness and boredom.^38^ Similarly, people with a strong sense of psychological entitlement, which refers to the feeling that they should be treated more favorably than others, were more likely not to cooperate with employee involvement.^40^, ^41^, ^42^ Therefore, they tended to be reluctant to comply with and participate in safety efforts.^43^ On the other hand, in collectivist cultures, workers found it difficult to openly share their concerns and ask for help because mentioning safety concerns was considered to be disruptive to the team and something to hide. This often led to stress and feelings of helplessness, which led to stagnation in safety compliance and participation.^44^

The reality of SMPs also varied by occupation.^45^ Workers who are unable to work from home, such as healthcare workers and frontline workers, have found it difficult to implement SMPs and therefore remain at high risk of COVID-19 infection.^46^, ^47^, ^48^ In addition, social distancing and other initiatives were difficult in interdependent workplaces that required face-to-face contact, workplaces that required frequent contact with customers, and workplaces where information technology was not widespread.^49^^,^^50^ As a result, workers in these occupational groups were more susceptible to fear of COVID-19 and psychological health disorders.^51^, ^52^, ^53^ In addition, organizations with many blue-collar employees and small organizations tended to be less active in COVID-19 safety compliance and participation owing in part to fewer financial resources available for the health of their members.^45^^,^^54^^,^^55^

On the basis of the above discussion, the following paragraphs discuss the factors that hinder or enhance employee involvement.

### Fear and Anxiety

The fear felt by members of the workplace during the COVID-19 pandemic is wide ranging, including not only the fear of themselves or their loved ones being infected but also the fear of infecting others, including colleagues. Excessive fear can lead to emotional fatigue among members, negatively impacting well-being, mental health, and work performance.^56^^,^^57^ In fact, fear of COVID-19 has been associated with health disorders and physical complaints, such as anxiety, fatigue, stress, depression, and insomnia.^58^^,^^59^ When employees have high emotional fatigue or a low level of mental health, even if the workplace provides a safe environment, morale and concentration decrease, and compliance with safety measures decreases.^60^^,^^61^ Moreover, because it was difficult to predict when the pandemic would end, people also had difficulty predicting what concrete rewards their efforts in controlling the infection would bring. When people fall into such a state of effort–reward imbalance, it becomes difficult for them to make the right decisions owing to the impairment of the reward network in the brain, centered on the striatum.^62^, ^63^, ^64^ The confusion and anxiety caused to employees in this way may impair safety compliance behavior by disrupting people's information-processing ability regarding health risks and leading them to make riskier decisions.^65^^,^^66^ Therefore, in a pandemic situation, fear and anxiety often led people to misbehave and make the workplace less safe, even though remaining calm and acting safely would be the more optimal choice.^67^^,^^68^

However, when organizations take preventive measures, the impact of fear and anxiety on increased emotional fatigue and decreased performance can be mitigated.^69^^,^^70^ In other words, promoting a safe environment and reducing emotional fatigue can enhance safety compliance behavior and health.^71^ Moreover, fear can benefit organizations if used well, because the fear they feel can be considered an adaptive response to perceived threats to maintain survival.^72^ Our bodies can respond quickly to dangerous situations by producing stress hormones that cause a fight or flight response when faced with stress.^73^ SMPs conducted by organizations during the pandemic had the effect of increasing employees' awareness of risk rather than reducing their fears related to COVID-19. This is because employees understood the dangers that COVID-19 poses at a global level by acquiring deeper knowledge about the COVID-19 pandemic through safety training and other means. Employees who are worried about their health or job stability tend to evaluate and analyze the situation according to protection motivation theory^16^^,^^17^ and act to protect themselves from threats. Therefore, even during the COVID-19 pandemic, employees who had fear or felt at risk performed safety compliance such as wearing masks, washing hands with soap or alcohol gel, and keeping at least 2 meters of interpersonal distance from others^74^^,^^75^ and also increased their work involvement through seeking support from social job resources, that is, supervisors and colleagues^76^ and engaged in OCB.^5^ In addition, employees who were concerned about receiving unfavorable evaluations from colleagues and supervisors if they were infected with COVID-19 in the organization were more proactive in increasing safety compliance behaviors.^77^, ^78^, ^79^, ^80^

Fear motivated not only employees but also their managers toward risk awareness and appropriate safety compliance behavior. If employees were to become infected with COVID-19 or lose motivation to work, organizations could face the risk of shutdown. Therefore, managers with higher risk awareness of COVID-19 were more likely to be proactive in safety compliance behavior.^81^ Moreover, leaders with high levels of positive emotions were able to use the fear generated by COVID-19 to improve their team’s goal achievement, commitment, and performance.^82^ Therefore, maintaining an appropriate level of worry and vigilance to maintain workplace safety during the COVID-19 pandemic is not something to be avoided but rather something to be consciously sought.^68^ However, it should be emphasized that such anxiety and fear are closely related to the risk of causing mental disorders such as stress, burnout, depression, and loneliness.^83^^,^^84^ Employees who experienced excessive stress and emotional fatigue owing to COVID-19 tended to have less energy, which negatively affected their safety compliance behavior.^67^^,^^68^ Moreover, SMPs implemented during the COVID-19 pandemic often exacerbated employees’ fears and anxieties, reducing their work performance.^85^ In particular, employees who were forced to work in unsafe conditions were at risk of experiencing a further decline in their mental health and well-being because SMPs stimulated their fears and anxieties about COVID-19.^24^

### Social and Psychological Resources

According to expectancy valence theory, individuals are more likely to behave safely if they believe that following safety standards will lead to better outcomes.^86^ Even in the workplace during the pandemic, employees were motivated to adopt safety compliance behaviors if they perceived that SMPs would lead to positive outcomes, such as reducing accidents and injuries.^44^^,^^87^ However, when the expectations formed were negative, they act to lower members' morale. As the pandemic continued, many employees observed others violating safety measures, such as not properly wearing face coverings, often in crowded spaces.^88^^,^^89^ Safety violations can endanger others around a person who committed the violation.^90^ In addition, frequently observing safety violations by others is experienced as a daily work stressor for employees.^91^ Employees who observe safety violations experience increased health anxiety and become nervous about their own health and safety. As a result, employees are more likely to divert attention from their duties^92^ or threats^91^^,^^93^ through work distractions.^93^^,^^94^ Work distractions are behaviors that reduce the time and effort put into work and contributions to the organization, such as repeatedly leaving work early or arriving late, or taking excessively long breaks.^95^ In addition, employees may quit work to avoid further threats or harmful situations.^77^^,^^96^, ^97^, ^98^ This makes SMPs more difficult as employee involvement decreases.

Once such a negative spiral has started, it is difficult to reverse it. However, Hobfoll’s^18^ conservation of resources theory can provide a clue to turn the situation around. According to the conservation of resources theory, employees have a caravan of resources and try to keep their amount constant. In the COVID-19 pandemic, if the resource of safety is maintained in the workplace through infection control measures, workers do not need to quit their jobs for fear of resource reduction.^89^^,^^98^ However, companies do not always have such resources. Especially in the case of unprecedented disasters such as COVID-19, companies do not know how to respond to maintain safety and, therefore, find themselves in a state of resource shortage. Hobfoll argues that when existing resources are not available, they can be replaced by social and psychological resources.^18^^,^^99^ Social resources or more commonly known as social capital primarily refers to the trust, norms, and networks that exist among members that promote cooperation and cohesion in organizations.^100^^,^^101^ As mentioned earlier, although SMPs have the effects of reducing the risk of infection, increasing the sense of security, and reducing the intention to leave the workplace, whether SMPs lead to actual safety depends on the social resources of the workplace.^102^^,^^103^ For example, at a Japanese local subsidiary in China, section managers communicated with employees every day through group chats to check whether their respective residences were under lockdown and to understand the situation, thereby reducing employees' anxiety and fear.^104^ When social resources are insufficient, employees may not trust their colleagues' infection control measures and may leave their jobs before the resources are depleted, even if they practice safety compliance behavior on the surface.^19^ On the other hand, psychological resources mainly refer to members' resilience and self-efficacy, which enable organizations to deal with difficulties and adapt to adversity.^105^, ^106^, ^107^ For example, at a Japanese subsidiary in Thailand, the memories and confidence of local employees from having overcome past flood damage served as psychological resources to enable them to think and act autonomously during the current pandemic, reducing employee anxiety and fatigue while also promoting infection prevention measures.^104^ Therefore, a lack of psychological resources hinders safety compliance and behavior^108^^,^^109^; increases emotional exhaustion^110^^,^^111^; and increases the job insecurity of employees with mental fatigue^112^ and depression,^113^ which has a negative impact on the health and behavior of anxious employees.^19^ These mechanisms are also applicable to the current workplace, where the pandemic is coming to an end and strict government restrictions on behavior have been lifted.^104^ Even today, the number of COVID-19 infections continues to increase, and the importance of resources remains, given that infection continues to result in people taking time off work and causing some disruption to organizational operations.

### Conservation of Resources Theory and Behavioral Theory

The formation of these resources may be related to behavioral theories. First, in social cognitive theory (SCT), which claims that cognitive factors, environment, and behavior interact to determine individual behavior, observational learning, which involves observing and learning from the behavior of others, plays an important role.^114^ Consistent with this theory, in a low-safety environment for COVID-19, observational learning may lead to moral disengagement and reduce preventive behavior.^115^ In addition, in accordance with the theory of planned behavior (TPB),^116^ 3 factors—subjective norm (a sense of moral obligation to perform or refrain from a certain behavior), attitude toward compliance behavior (an individual's evaluation of the planned behavior), and sense of behavioral control (an individual's perception of their ability to perform a certain behavior)—influenced compliance with COVID-19 prevention guidelines.^117^ Finally, in accordance with the health belief model (HBM), a widely used behavioral change theory for predicting health-related behaviors,^118^ the perceived benefits of infection prevention promoted compliance intentions, whereas perceived barriers reduced compliance intentions.^119^ That is, SCT claims that increasing the workplace safety environment, TPB claims that increasing the expectations of others and perceptions of self-efficacy regarding infection control, and HBM claims that public awareness of the benefits of infection prevention behaviors and the provision of necessary resources are factors that increase compliance. However, by combining these behavioral theories with resource conservation theory, organizations may further enhance their understanding and resilience to disasters. For example, SCT's moral disengagement may arise from observational learning in an organization lacking social resources, and the disengagement may be further promoted by others imitating the disengagement. The subjective norm of the TPB is related to the degree of obligation to participate in infection control measures in the workplace, and the attitude of the TPB is related to whether there are benefits to participating in infection control measures, so it depends on the size of social resources in the workplace and may contribute to the formation of social resources in the workplace. On the other hand, the sense of behavioral control of the TPB is related to confidence in being able to carry out infection control measures and may have a mutually enhancing relationship with both psychological and social resources. Finally, the HBM suggests that when psychological and social resources are abundant, employees are more likely to perceive the benefits of infection prevention measures and less likely to perceive barriers, making it easier for them to practice health behaviors.

## DISCUSSION

The pandemic has provided lessons for workplace health management. Organizations have had to implement SMPs that include safety initiatives and employee involvement in line with their OSHMS in response to COVID-19. In addition, methods to increase employee involvement include fear and anxiety as well as social and psychological resources. There is a certain rationality in encouraging fear and anxiety in keeping workplaces safe during crises. However, continuing to rely on employees' fight-or-flight instincts can wear them down and lead them to adopt undesirable behaviors for the organization, such as quitting their jobs. To avoid this, it is necessary to develop social and psychological resources. However, previous research has only emphasized the importance of these resources, and there has been little discussion about how to develop them. Some studies suggest that these resources may be maintained or strengthened rather than diminished by disasters. For example, recent studies have shown that people not only felt hurt and stressed during the COVID-19 pandemic but also maintained or strengthened positive attitudes as they weathered the crisis.^120^^,^^121^ Another study showed that the COVID-19 pandemic had a positive impact on all areas of safety culture, namely the physical environment, employee behavior, and internal characteristics of employees, with the greatest improvement being in the area of concern for one's own safety and the safety of colleagues.^122^ The persistence and universality of the resource effect shown in one study^104^ may be well explained by the beneficial side effect of the pandemic, but of course, disasters cannot and should not be artificially caused. Future research should focus on identifying ways to strengthen employees' social and psychological resources without relying on disasters. To achieve this, research that combines conservation of resources theory with major behavioral theories and aims to develop more specific, actionable methods for cultivating resources is needed. In addition, as the methods for implementing this in society include, for example, an approach that leverages the immersive potential of augmented reality and virtual reality simulations to reflect the complexity of real-work disaster scenarios could be developed. To achieve this, discussions in business administration and public health need to be effectively bridged and utilized with other fields such as engineering. It is hoped that this review will be of some help in this regard.

Previous studies have provided evidence suggesting that countries with more severe disaster experience were more likely to have paid greater public attention to the COVID-19 pandemic response, more timely market responses, and stricter government containment measures.^123^ However, other studies argue that the pandemic is a unique disaster that has little to do with other disaster types and requires a completely different planning approach.^124^ The reason, they argue, is that whereas in other disasters, public trust and behavior have less of an impact on the authorities' overall ability to manage the situation, the lack of public trust during COVID-19 worsened the situation by increasing the number of cases and deaths, which in turn led to a decline in the resilience of the population.^124^ If this is the case, then the success or failure of the response to COVID-19 can be evaluated as being unique, supported by people's social and psychological resources. At the workplace level, there have been detailed reviews of changes in working patterns, such as an increase in remote work, due to the COVID-19 disaster experience,^125^ but there is a lack of discussion about how specific workplace-level responses during the pandemic can be utilized in future disasters. In this context, 1 study has shown that social workers may have increased their risk awareness and motivation to work on pandemics and natural disasters.^126^ Thus, even if COVID-19 is a unique disaster, there may have been changes in workers' disaster awareness that should be inherited.

For example, when an unprecedented crisis such as COVID-19 occurs, managers can immediately take measures such as restricting attendance to prevent the spread of infection to employees. However, doing so may cause production activities to halt, and there is a risk of losing revenue that would have been obtained if production had continued. Of course, in the medium term, companies will follow the decisions of the government in the country where they have expanded and the instructions of the parent company in their home country, but in the meantime, individual companies have discretion in how they judge and act. Therefore, in companies that can respond quickly, employees will feel reassured and increase their commitment to the workplace, whereas in companies that respond slowly, employees will become more anxious and consider leaving their jobs. Therefore, it is necessary for individual workplaces to take steps to pass on these experiences in concrete terms, rather than leaving them as tacit knowledge, to face the next disaster with confidence. However, in workplaces with low employee retention rates or where top management is frequently replaced, such as overseas local subsidiaries, such experiences may not be passed on, and it may be difficult to utilize them. Therefore, even if other disaster experiences are utilized during the COVID-19 pandemic, there may not be much optimism about the extent to which the experience of the COVID-19 pandemic, which relied heavily on the utilization of invisible, social, or psychological resources, can be applied to future disaster experiences. In the future, evidence on how the experience of the COVID-19 pandemic is utilized in other disasters should be collected and actively discussed. This could increase the significance of this disaster in public health and strengthen preparations for future disasters.

## CONCLUSIONS

The pandemic has provided lessons for workplace health management. Organizations have had to implement SMPs that include safety initiatives and employee involvement in line with their OSHMS in response to COVID-19. In addition, methods to increase employee involvement include fear and anxiety as well as social and psychological resources. Fear and anxiety promote the practice of safety compliance behavior, but they also wear down employees and increase their work distraction and willingness to leave. Therefore, even if the method of encouraging employees to practice safety compliance behavior by inciting fear and anxiety has a certain beneficial effect, in the long term, it will weaken the organization. To overcome this dilemma, it is necessary to strengthen social and psychological resources so that employees are convinced that safety compliance and the practice of safety behavior will lead to a safe workplace and will be actively involved. These resources can also be used for infection prevention measures today, when the pandemic has subsided and fear and anxiety are no longer motivating employees. To this end, research is needed to improve organizations' understanding of disasters and their resilience by integrating conservation of resource theory and behavioral theory as well as the development of virtual technologies that can concretely imagine and respond to problems that may arise during disasters.
